# Involvement of Innate and Adaptive Immune Systems Alterations in the Pathophysiology and Treatment of Depression

**DOI:** 10.3389/fnins.2018.00547

**Published:** 2018-08-17

**Authors:** Eva M. Medina-Rodriguez, Jeffrey A. Lowell, Ryan J. Worthen, Shariful A. Syed, Eléonore Beurel

**Affiliations:** ^1^Department of Psychiatry and Behavioral Sciences, Miller School of Medicine, University of Miami, Miami, FL, United States; ^2^Department of Biochemistry and Molecular Biology, Miller School of Medicine, University of Miami, Miami, FL, United States

**Keywords:** GSK3, innate immune response, adaptive immune response, depression, NF-κB

## Abstract

Major depressive disorder (MDD) is a prevalent and debilitating disorder, often fatal. Treatment options are few and often do not provide immediate relief to the patients. The increasing involvement of inflammation in the pathology of MDD has provided new potential therapeutic avenues. Cytokine levels are elevated in the blood and cerebrospinal fluid of MDD patients whereas immune cells often exhibit an immunosuppressed phenotype in MDD patients. Blocking cytokine actions in patients exhibiting MDD show some antidepressant efficacy. However, the role of cytokines, and the immune response in MDD patients remain to be determined. We reviewed here the roles of the innate and adaptive immune systems in MDD, as well as potential mechanisms whereby the immune response might be regulated in MDD.

## Introduction

Once considered primarily as a component of the body’s responses to injury and infection, inflammation is now also known to be an important component of neuronal function and dysfunction. Here we consider recent evidence of these roles of inflammation and its potential as a therapeutic target in major depressive disorder (MDD). MDD is a heterogeneous, recurrent, and debilitating disease that has a high prevalence worldwide, has unknown etiology, and is inadequately treated in approximately two-thirds of patients (Rush et al., 2006). During the last two to three decades inflammation has emerged as an important factor contributing to MDD (Miller and Raison, 2016) and evidence is emerging that it may provide a new target for therapeutic intervention.

## Immune System and Depression

### The Innate and Adaptive Immune Systems

The immune system is a complex network of many types of immune cells and secreted factors that contribute to regulating inflammation in all tissues of the body. The immune system constantly surveys its environment to ensure health and is activated following infection for pathogen clearance, following tissue damage to support repair and by psychological stress.

The immune system is commonly characterized as consisting of two arms: the innate immune system and the adaptive immune system, even though both the innate and adaptive immune systems communicate and cooperate with each other to direct pro- and anti-inflammatory responses with the main goals of eradicating pathogens and restoring homeostasis. Cytokines and chemokines, which are produced during innate and adaptive immune responses, are thought to mediate some of these effects, particularly by mobilizing and modulating the function of immune cells. The innate immune system composed of antimicrobial peptides and myeloid lineage cells (e.g., monocytes/macrophages, dendritic cells, neutrophils, and others) is the first line of response to pathogens or insults. Innate immune cells are sentinels that constantly survey the environment for non-self signals, using pattern-recognition receptors [e.g., Toll-like receptors (TLRs)] that recognize common microbial features (virus, bacteria, fungi, parasites) or other damage signals. If the innate immune system fails to resolve the insult, a second line of defense by the adaptive immune system is activated. While the innate immune response occurs quickly, the adaptive immune response, composed of lymphocytes and secreted antibodies, relies on the proliferation and differentiation of antigen-specific T cells and B cells that requires time to be mounted (den Haan et al., 2014). The advantage of the adaptive immune response is the selectivity for antigen, since the T cell-receptor and B cell-receptor systems provide a pathogen-tailored immune response. In addition, the adaptive immune system is thought to be responsible for immunological memory which is critical in the case of re-exposure to the same antigen.

It is important to note that within each organ, there are also often specialized immune cells, such as microglia in the central nervous system (CNS). Microglial cells derive from a myeloid lineage in early development when they colonize the neural tube. It was thought for a long time that microglial cells were the only immune competent cells to mediate immune responses in the CNS because the CNS was thought to be isolated from the immune system by the presence of the blood–brain barrier (BBB) that prevents the free entry of immune cells into the CNS. However, recent evidence shows that immune cells can infiltrate the CNS (Korn and Kallies, 2017). In addition, stress, the most common factor that precipitates depressive episodes, has been shown to induce inflammatory responses both in the brain and in the periphery by activating similar pathways (e.g., TLRs) that are activated by pathogens on innate immune cells. Here, we review the role of both the innate and adaptive systems in MDD and their potential contributions to disease.

### Roles of the Innate Immune System in Mouse Models of Depression and in MDD Patients

A diverse array of evidence has been reported implicating the innate immune system involvement in the pathology of MDD (Hodes et al., 2015). It has been known for decades that MDD patients often have elevated levels of circulating granulocytes and monocytes (Maes et al., 1992; Maes, 1995; Seidel et al., 1996b), pro-inflammatory cytokines and their receptors, acute phase proteins, chemokines, and prostaglandins (Miller et al., 2009), indicative of activation of the peripheral immune system in MDD. Similarly, an increased number of circulating monocytes has been observed in mice exhibiting depressive-like behaviors (Engler et al., 2004) and neutralization of monocytes is sufficient to reduce depressive-like behaviors (Zheng et al., 2016). While examining the mechanisms whereby monocytes promote depressive-like behaviors, it has been proposed that this is due to secretion by monocytes of cytokines that are capable of promoting depressive-like behaviors (Miller et al., 2009). Furthermore, activated peripheral monocytes traffic to the brain vasculature and parenchyma during the induction of depressive-like behavior, triggering inflammation in the CNS and therefore contributing to the pathophysiology of depression (Wohleb et al., 2013). Monocytes infiltrate and colonize brain regions associated with depression and anxiety in mice exposed to social defeat stress (Sawicki et al., 2015). Conversely, depressive-like behavior induced following peripheral organ inflammation is reversed by blocking the brain infiltration of monocytes (D’Mello et al., 2009). This points out the importance of the influx of peripheral innate immune cells such as monocytes into the brain to maintain depressive-like behaviors. Among the mechanisms proposed for the increase of brain monocytes after stress is increased release of bone marrow derived monocytes to the bloodstream after stimulation by catecholamines produced by the sympathetic nervous system after stress (Wohleb et al., 2011, 2013; Heidt et al., 2014). Once released, monocytes are recruited to the brain upon receiving signals secreted by activated microglia, including two key chemokine receptors, chemokine receptor-2 (CCR2) and fractalkine receptor (CX3CR1) (D’Mello et al., 2009; Wohleb et al., 2013). Once in the brain, monocytes recruited by microglial cells are thought to produce interleukin (IL)-1β, which promoted anxiogenesis through the activation of neurovascular IL-1R1 (McKim et al., 2017). Consistent with the presence of monocytes in the brain of mice exhibiting depressive-like behaviors, post-mortem analyses showed increased numbers of perivascular tissue resident macrophages associated with elevated levels of the C–C chemokine ligand 2 (CCL2) in the brain of MDD patients (Torres-Platas et al., 2014). These and other studies indicate that peripheral innate immune cells are activated and reach the brain in depression and animal models of depression.

### Role of Astrocytes and Microglia in Mouse Models of Depression and in MDD Patients

Both astrocytes and microglia have been implicated as contributing to the pathophysiology of depression. MDD is associated with loss and hypotrophy of astrocytes (Davis et al., 2002; Miguel-Hidalgo et al., 2010). This mainly occurs in the frontolimbic systems that are relevant for major depression (Ongur et al., 1998; Rajkowska et al., 1999; Miguel-Hidalgo et al., 2000; Cotter et al., 2002; Altshuler et al., 2010; Gittins and Harrison, 2011; Rajkowska and Stockmeier, 2013; Torres-Platas et al., 2014; Nagy et al., 2015; Rial et al., 2015; Cobb et al., 2016; Medina et al., 2016; Rubinow et al., 2016). Interestingly, there is no astrogliosis in MDD patients, which contrasts with the prominent astrogliosis that commonly occurs in patients with neurodegenerative diseases. Loss and shrinkage of astrocytes also have often been reported in mouse models of depression (Czeh et al., 2006; Rajkowska and Stockmeier, 2013; Sanacora and Banasr, 2013). That reduced astrocyte functions may contribute to depression is supported by the finding that elimination of astrocytes from the rat prefrontal cortex (PFC) is sufficient to induce depressive-like behaviors (Banasr and Duman, 2008). Antidepressant treatments reverse these stress-induced astrocytic morphological changes to restore astrocytic function, as well as reverse associated depressive-like behaviors (Rajkowska and Stockmeier, 2013; Rial et al., 2015). These findings support the current theory that astrocytic dysfunction contributes to the pathophysiology of MDD and that the cellular actions of antidepressants may correct or compensate for impaired function of astrocytes (Manji et al., 2003; Czeh et al., 2006).

Promotion of depression by impaired astrocyte function may be due to consequential neuronal damage, including induction of neuronal excitotoxicity, because of the roles of astrocytes in supporting neuronal function by the uptake of synaptic glutamate or the production of growth factors (Sofroniew and Vinters, 2010). Consistent with this, astrocyte density (Miguel-Hidalgo et al., 2000) and synapse-related genes (Kang et al., 2012) are both decreased in the dorsolateral PFC of MDD patients. In addition, knockout of the astrocytic glutamate transporter GLT-1, which is important for the reuptake of extracellular glutamate, in the lateral habenula of mice, is sufficient to induce depressive-like behaviors (Cui et al., 2014). Altogether, these findings suggest that loss of astrocytes might promote depression by impairing neuronal functions.

Many other functions of astrocytes are altered in depression, including formation of gap junctions, neurotrophic support, energy metabolism, water homeostasis, γ-aminobutyric acid (GABA) and monoamine recycling, and gliogenesis, and antidepressant treatments tend to reverse most of these alterations (Tavares et al., 2002; Simard and Nedergaard, 2004; Czeh et al., 2006; Haydon and Carmignoto, 2006; Hundal, 2007; Rouach et al., 2008; Eulenburg and Gomeza, 2010; Sofroniew and Vinters, 2010; Cao et al., 2013; Jiang and Salton, 2013; Martin et al., 2013; Rajkowska and Stockmeier, 2013; Rajkowska et al., 2013; Nagy et al., 2015; Rial et al., 2015; Bjorkholm and Monteggia, 2016). Therefore, it remains to be determined by which mechanism(s) astrocytic atrophy promotes depression. In addition, it is unclear what causes astrocytic atrophy in MDD patients and whether this atrophy results from an inflammatory process in the brain mediated by immune cells.

Microglial cells have been shown to be activated in various brain regions of MDD patients, including in the dorsolateral PFC, anterior cingulate cortex, mediodorsal thalamus, insula and hippocampus, both in post-mortem tissue and by positron emission tomography (PET) imaging (Steiner et al., 2008; Schnieder et al., 2014; Setiawan et al., 2015). Activation of microglia often is the consequence of increased inflammation in the brain (Dantzer et al., 2008; Rivest, 2009; Benarroch, 2013; Slavich and Irwin, 2014; Delpech et al., 2015; Jo et al., 2015; Ransohoff et al., 2015; Menard et al., 2016; Miller and Raison, 2016; Wachholz et al., 2016; Haroon et al., 2017). Characterization of microglia in mouse models of depression showed that microglial cells adopt an activated phenotype expressing surface markers such as major histocompatibility complex (MHC) class II, CD86 or CD54 (Wachholz et al., 2016) and producing pro-inflammatory cytokines [e.g., IL-1β, IL-6, tumor necrosis factor α (TNFα)] (Chabry et al., 2015), toxic molecules (e.g., nitric oxide) or extracellular vesicles, such as exosomes, which might be responsible for propagating inflammatory signals throughout the brain (Fruhbeis et al., 2013). Microglial cells, once activated, are often eliminated (Kreisel et al., 2014). In a model of unpredictable chronic stress, it was shown that after a first phase of microglial activation, microglial cells decrease in number in the hippocampus of stressed mice, whereas the blockade of microglia activation was sufficient to prevent the reduction in microglia number, and the induction of unpredictable chronic stress-associated depressive-like behaviors (Kreisel et al., 2014). In contrast, neuroprotective microglia diminish in the hippocampus (Branchi et al., 2014), PFC (Hinwood et al., 2012), and amygdala (Hamidi et al., 2004) after chronic stress.

Microglial cells express several inflammatory pathways that are responsible for cytokine production, including TLR pathways. Microglial cells are the main producers of cytokines in the brain after exposure to stress. In response to stress, microglial cells are activated by molecules called alarmins, danger-associated molecular pattern molecules, or damage-associated molecular pattern molecules. For example, the protein high mobility group box-1 (HMGB1) is up-regulated and it activates the TLR4 and receptor for advanced glycation end-products (RAGE) pathways resulting in increased production of proinflammatory cytokines (Weber et al., 2015; Franklin et al., 2018). Central administration of HMGB-1 also induces depressive-like behaviors (Wu et al., 2015; Cheng et al., 2016; Franklin et al., 2018), altogether suggesting that up-regulation of alarmins such as HMGB1 contribute to the development of depressive-like symptoms by activating microglial cytokine production. Additionally, cytokines, such as TNFα, also can disrupt BBB permeability, raising the possibility that increased BBB permeability caused by microglia-derived cytokines contributes to depression (Menard et al., 2017; Cheng et al., 2018) by promoting the infiltration of peripheral inflammatory molecules and immune cells (Wohleb et al., 2011, 2013).

Microglial cells contribute to synaptic pruning by phagocytosis of synapses during development but also possibly during adulthood (Kettenmann et al., 2013; Miyamoto et al., 2013; Wake et al., 2013; Wohleb et al., 2018). Therefore microglia can modulate neuronal circuits (Wake et al., 2013), and these processes can increase the production of neurotrophic factors in response to neuronal stimulus (Nakajima et al., 2007). Therefore, microglia activation has direct effects on neuronal function. Microglia activation impedes microglia-neuron communication via the CX3CR1-fractaline pathway after chronic stress (Corona et al., 2010; Milior et al., 2016). Furthermore, stress-induced anxiety- and depressive-like behaviors are associated with increases in neuronal colony stimulating factor 1 (CSF1) which activates microglial phagocytosis of synaptic elements in the medial PFC. This demonstrates the importance of reshaping neuronal connectivity by the neuronal-activated microglia communication in promoting depressive-like behaviors (Wohleb et al., 2018). Moreover, microglial activation after stress-induced depressive-like behavior induces the activation of the kynurenic acid pathway (Jo et al., 2015), leading to the depletion of monoamines and the production of quinolinic acid (Steiner et al., 2011), and ultimately to the dysregulation of monoaminergic and glutamatergic circuits (Jo et al., 2015). Consistent with the fact that microglial cells are particularly enriched in the hippocampus, a brain region relevant for stress response and neuroplasticity, the role of microglia appears to be critical for maintaining neuronal homeostasis and neurotransmission, whereas activation of microglia by stress alters this equilibrium, which can promote susceptibility to depressive-like behaviors.

### Role of the Adaptive Immune System (T Cells and B Cells) in Mouse Models of Depression and in MDD Patients

In addition to increased peripheral monocytes, atrophy of astrocytes, and increased activation of microglia, MDD patients also exhibit decreased numbers of circulating T cells and a reduction in regulatory B cells (Ahmetspahic et al., 2018), suggestive of a dysregulation of the adaptive immune system in MDD patients. In addition, T cells from MDD patients have a reduced capacity to respond to stimulation, indicative of an immunosuppressed phenotype of T cells in MDD (Herbert and Cohen, 1993; Zorrilla et al., 2001; Irwin and Miller, 2007). Besides a global immunosuppression of T cells in MDD, there are also alterations of the T helper (Th) (CD4^+^) and T cytotoxic (CD8^+^) cells in MDD patients. Both Th (CD4^+^) and T cytotoxic (CD8^+^) cells have altered activities in MDD patients exemplified by an increase of the ratio of CD4^+^ T cells relative to CD8^+^ T cells, suggestive of an increase of CD4^+^ cells and/or a decrease of CD8^+^ cells favoring the Th cells. Differentiation of the different subsets of CD4^+^ Th effector cells, Th1, Th2, Th17, and regulatory T cells (Tregs) require specific cytokines, antigen presentation, and co-stimulatory signals for the production of each subtype. Evaluation of the differentiation status revealed a decrease in circulating Tregs (reviewed in Li et al., 2010; Martino et al., 2012; Toben and Baune, 2015) and an increase in the Th1/Th2 ratio (Myint et al., 2005), whereas circulating Th17 cells are increased in MDD patients (Chen et al., 2011). These findings indicate that there is a massive remodeling of the Th compartment in MDD patients that might be dictated by the cytokine milieu present in MDD patients. The exacerbation of the Th2 responses in MDD or asthma patients might contribute to the higher rate of both MDD in asthma patients (Van Lieshout et al., 2009), and asthma in MDD patients (Shen et al., 2017). However, further studies are required to identify the underlying mechanisms. Mice exhibiting depressive-like behaviors also show increased levels of brain Th1 and Th17 cells (Beurel et al., 2013). Although the contribution of these various Th cells to MDD pathology remains to be determined, it is worth noting that administration of Th17 cells is sufficient to promote depressive-like behaviors in mice (Beurel et al., 2013). Recent findings revealed that the Th17 cells that accumulate in the brains of mice exhibiting depressive-like behavior express characteristics of both pathogenic Th17 cells and of follicular T cells (Beurel et al., 2018). To further corroborate the notion that T cell alterations contribute to MDD, antidepressant treatments have been shown to restore the Th1/Th2 imbalance in MDD patients (Martino et al., 2012), which may be mediated by increasing Treg levels (Himmerich et al., 2010). Together these findings suggest that T cell alterations are present in MDD patients and might contribute to the development and maintenance of MDD.

The role of T cells in modulating mood and behaviors has mainly been studied in the context of examining the effects of the absence of T cells using lymphopenic mice devoid of both T cells and B cells. T cells have been shown to be beneficial to brain function through neuroprotective roles (Miller, 2010; Filiano et al., 2017), providing continual brain immune surveillance (Lewitus et al., 2008), and promoting neurogenesis (Ziv and Schwartz, 2008), cognition (Schwartz and Kipnis, 2011), and mood (Herkenham and Kigar, 2017). Thus, CNS-targeted T cells promote brain health and function, supporting the notion of “protective autoimmunity” (Rook and Lowry, 2008; Lewitus and Schwartz, 2009; Lewitus et al., 2009). Similarly, immunization of mice with a modified CNS antigen to generate weak CNS-autoreactive T cells protected mice from the development of depressive-like behavior following stress (Cohen et al., 2006; Lewitus et al., 2009). However, T cells may not require CNS-specificity to be beneficial to the CNS, as adoptive transfer of lymphocytes to lymphopenic mice rescues sociability deficits displayed by lymphopenic mice (Filiano et al., 2016) and confers antidepressant-like effects (Clark et al., 2016). These results suggest that T cells contribute to normal affective behavior in mice by supporting an adaptive response to stress (Herkenham and Kigar, 2017). Furthermore, administration of splenocytes from socially defeated mice, but not from unstressed mice, protects lymphopenic recipient mice from the effects of social defeat and confers both anxiolytic and antidepressant-like effects (Brachman et al., 2015; Scheinert et al., 2016), suggesting that stress may produce changes in T cells that promote resilience to future stress. These beneficial effects of T cells contrast with the deleterious effects of T cell subsets in autoimmune diseases, where subsets of T cells drive pathogenicity. It is possible that in depression, similar deleterious roles of T cell subsets exist, whereas most of the studies have been focused on the overall importance of T cells in mediating depressive-like behaviors using lymphopenic mice.

## Cytokines In Depression and Pathways Controlling Inflammation In Depression

### Overview of Meta-Analyses of Cytokine Levels in Depressed Patients Compared to Healthy Controls

Major depressive disorder (MDD) has been associated with alterations of both the innate and the adaptive immune responses (Dantzer, 2009) and MDD patients often experience elevated levels of cytokines (Felger and Lotrich, 2013). Although it is difficult to find a consensus in the many different cytokines that have been reported to be altered in MDD patients in various studies, the levels in both blood and cerebrospinal fluid (CSF) of TNFα, IL-6 and IL-1β have very often been found to be significantly elevated in individuals with MDD compared to healthy controls (Maes et al., 1997; Levine et al., 1999; Tuglu et al., 2003; O’Brien et al., 2004; Howren et al., 2009; Lindqvist et al., 2009; Dowlati et al., 2010; Hiles et al., 2012; Haapakoski et al., 2015; Kohler et al., 2017). In addition to these three pro-inflammatory cytokines, elevated levels of several anti-inflammatory cytokines [IL-2, IL-4, IL-10, transforming growth factor β (TGFβ)] have also been associated with MDD (Kubera et al., 2001b; Hernández et al., 2008; Sutcigil et al., 2008; Fazzino et al., 2009), whereas other cytokines, such as sIL-2R, CCL-2, IL-13, IL-18, and IL-12, have been found to be elevated in the blood of MDD patients in a few studies (Maes, 1995; Kim et al., 2002; Merendino et al., 2002; Pavón et al., 2006; Köhler et al., 2017). In addition, the acute phase protein, C-reactive protein (CRP), which is an innate immune marker reflecting peripheral inflammation, correlates with central inflammation, and is associated with depressive symptom severity (Felger et al., 2018). Overall, these findings suggest a general induction of cytokine production in MDD patients. However, not all cytokines have been shown to be elevated in MDD. For example, interferon (IFN)γ was often found to be lower in MDD patients compared to healthy controls (Hernández et al., 2008; Fornaro et al., 2013; Ho et al., 2015). In addition to being elevated in the blood and CSF of MDD patients, there is also evidence that cytokines elevated in the brains of MDD patients. Post-mortem measurements of brain tissue samples from Broadman area 10 (frontal cortex) showed significant increases of IL-1α, IL-2, IL-10, and IFNγ mRNA levels in suicide completers (Shelton et al., 2011).

The presence of elevated levels of cytokines in MDD patients raises the question of whether inflammation causes depression or is a consequence of the disease. The literature suggests a bidirectional relationship between depression and inflammation: inflammation is capable of inducing depressive-like symptomatology and depressive-like behavior is associated with elevated cytokine levels (Dantzer, 2009). Thus, for example, elevation of certain cytokines has been associated with the severity of MDD (Lindqvist et al., 2009) and in a prospective study of geriatric patients with no psychiatric history, the elevation of cytokines preceded the onset of depressive symptoms (Baune et al., 2012). In the Avon Longitudinal Study of Parents and Children birth cohort, higher serum concentration of IL-6 at 9 years old predicted the likelihood of developing MDD symptoms at 18 years old (Khandaker et al., 2014) and another longitudinal study found a similar association (Gimeno et al., 2009). Consistent with the notion that a low grade inflammation might increase susceptibility to MDD, healthy volunteers receiving a low dose of lipopolysaccharide (LPS), which triggers cytokine production, exhibit depressive symptoms whose severity correlated with cytokine production (Reichenberg et al., 2001). Furthermore, IFNα infusion therapy is associated with 30–50% of patients developing depression, in a dose-dependent manner (Capuron et al., 2005). Moreover, many inflammatory diseases, such as multiple sclerosis, are co-morbid with depression with an estimation of 60–80% of multiple sclerosis patients exhibiting depression (Caine and Schwid, 2002; Pucak et al., 2007). Other diseases with an inflammatory component such as diabetes mellitus (Anderson et al., 2001) and coronary artery disease (Rudisch and Nemeroff, 2003) have been shown to have a high prevalence of comorbidities with depression. Similar findings were reported in mice where higher inflammation is associated with depressive-like behaviors (Henry et al., 2008; O’Connor et al., 2009). Additionally, psychological stress (Maes et al., 1998) that often triggers depression is sufficient to induce pro-inflammatory responses. Taken together, depression is accompanied by significant changes of cytokine production which indicate activation of the inflammatory response in MDD patients, and inflammation appears to promote susceptibility to depression as well as to be induced by depression.

### Mechanisms Regulated by NF-κB and GSK3 Contributing to Inflammation in Depression

Although changes in cytokine production in MDD are well-established, the mechanisms whereby these cytokines are produced in MDD patients remain largely unknown. Multiple mechanisms likely contribute to inflammation in depression. Mechanistic studies in rodents have shown the importance of a few key pathways in controlling cytokines production. Among these, here we will focus on nuclear factor-κB (NF-κB) and glycogen synthase kinase 3 (GSK3) pathways, which are predominant molecular pathways regulated by stress.

#### TLR4/GSK3/NF-κB

One of the major pathways regulating the production of most cytokines after infection, injury or stress is the TLR4 pathway. TLR4 is a pattern-recognition receptor that leads to activation of the transcription factor NF-κB to increase the production of many cytokines. TLR4 is activated following recognition of microbial peptides (such as LPS) after infection or endogenous molecules (HMGB1, heat shock proteins, ATP) that are released after cellular damage or psychological stress. Although TLR4s are most well-recognized as being expressed by innate immune system cells, they are also expressed by neurons and glia (Slavich and Irwin, 2014). Altogether 10 functional human TLRs have been identified (12 in mice) and among these, TLR4 has been most repeatedly linked with the induction of inflammation following psychological stressors (Jope et al., 2017). TLR4 activation up-regulates its own expression, and elevations of TLR4 mRNA and protein have been detected in both the periphery and CNS of MDD patients (Hung et al., 2014). Furthermore, successful treatment of MDD restores levels of TLR4, pointing toward a role for TLR4 in MDD patients (Raison and Miller, 2017). In addition, TLR4 ligands (e.g., HMGB1) are induced by stress in animal models of depression (Fleshner, 2013). Stress has also been shown to promote NF-κB activation, which likely results at least in part from activation of TLR4 (Cheng et al., 2016). Another important link in the TLR4 pathway is GSK3. Indeed, TLR4-mediated cytokine production is highly dependent on active GSK3 (Martin et al., 2005). GSK3 promotes NF-κB activation and NF-κB-dependent production of proinflammatory cytokines, whereas GSK3 inhibits cAMP response element binding (CREB)-dependent production of anti-inflammatory cytokines (Martin et al., 2005), so GSK3 activation, which occurs after many types of stress, favors proinflammatory responses. Furthermore, GSK3 is activated in post-mortem brains from MDD patients and in mice exhibiting depressive-like behaviors (Beurel, 2011; Beurel et al., 2015), and most antidepressant drugs inhibit GSK3 (Beurel et al., 2015). In addition, GSK3 promote IFNγ-dependent indoleamine 2,3-dioxygenase (IDO) expression by modulating the Janus kinase (JAK)/signal transducer and activator of transcription (STAT) pathway in dendritic cells (Noh et al., 2015). This might also be mediated by NF-κB because non-canonical NF-κB signaling is required to induce IDO in dendritic cells by modulating the JAK/STAT pathway (Tas et al., 2007). Altogether this suggests another potential role of both GSK3 and NF-κB in promoting depressive-like behavior by modulating IDO. Therefore, GSK3 is an important hub linking stress, inflammation, and depression via the TLR4–GSK3–NF-κB signaling axis.

#### Inflammasome

The inflammasome pathway is selectively responsible for the production of IL-1β and IL-18 and is part of the innate immune response that contributes to cytokine production. It is a cytosolic complex, composed of Nod-like receptor (NLR), caspase-1, and apoptosis-associated speck-like protein containing C-terminal caspase recruitment domain (ASC)-1. Once activated, this complex leads to the caspase-1-mediated cleavage of pro-IL1β or pro-IL-18 to produce active IL-1β and IL-18 (Fleshner et al., 2017). Among the NLR subtypes, the NLR protein 3 (NLRP3) inflammasome has often been studied in the context of CNS inflammation related to stress and depression. The NLRP3 inflammasome requires a two-step process to produce cytokines, consisting first of the assembly of a primed inflammasome with procaspase-1 followed by activation of the inflammasome involving cleavage, and thereby activation, of procaspase-1. Thus, HMGB1, for example, induces the assembly of the NLRP3 inflammasome by activating TLR4 (Fleshner et al., 2017). The NLRP3 inflammasome is then activated by another or the same signal (e.g., HMGB1, ATP, heat-shock proteins) (Miller and Raison, 2016; Fleshner et al., 2017). NLRP3- and caspase-1-deficient mice are resilient to depressive-like behavior, indicating that inflammation caused by activation of the NLRP3 inflammasome regulates susceptibility to depression-like behaviors (Alcocer-Gomez et al., 2016; Iwata et al., 2016; Wong et al., 2016). In contrast, antidepressant drugs prevent inflammasome activation (Alcocer-Gomez et al., 2017). In addition, MDD patients have increased expression of NLRP3 and caspase-1 in circulating immune cells, suggesting that the NLRP3 inflammasome is activated in MDD patients, which correlates with increased levels of IL-1β and IL-18 in MDD patients (Alcocer-Gomez et al., 2014). Interestingly, the inflammasome assembly is controlled by both NF-κB and GSK3 (Guo et al., 2015; Jope et al., 2017). Thus, the inflammasome is primed by a NF-κB-dependent signal (Guo et al., 2015). Moreover, in lupus nephritis, GSK3 activates NLRP3 and therefore, IL-1β production, and psychological stress activating GSK3 induces the NLRP3/IL-1β pathway (Liu et al., 2015; Cheng et al., 2016). It remains to be determined if GSK3 actions on the inflammasome are NF-κB dependent.

#### HPA Axis

The hypothalamus–pituitary–adrenal (HPA) axis activation is the main response to stress. It is a complex interaction between the paraventricular nuclei of the hypothalamus, the pituitary gland, and the adrenal glands, a major neuroendocrine system that controls many functions, such as digestion, immune responses, and mood and emotions. In response to stress, corticotropin-releasing factor (CRF) is released, which initiates a cascade of events comprising the release of adrenocorticotropic hormone (ACTH) leading to the production of glucocorticoids (cortisol in humans, corticosterone in rodents) from the adrenal glands (Herkenham and Kigar, 2017). The HPA axis is finely tuned in healthy individuals via negative-feedback since its activation leads to profound metabolic, physiologic, and immunologic changes across nearly all body systems, including the brain (Sapolsky et al., 2000; Herbert et al., 2006; Silverman and Sternberg, 2012). Thus, glucocorticoids regulate the magnitude and duration of the glucocorticoids release. In addition, the HPA axis receives feedback from afferent projections from various brain regions, such as brain stem noradrenergic neurons. Glucocorticoids bind to the glucocorticoid receptor (GR), which is part of a multiprotein complex consisting of several heat shock proteins. Once activated upon binding of its ligand, the GR dissociates from the heat shock protein complex to migrate to the nucleus where it binds to DNA binding elements or interacts with other transcription factors, such as NF-κB. Two-thirds of MDD patients have elevated levels of circulating glucocorticoids (Stetler and Miller, 2011), and genetic studies have identified polymorphisms and epigenetic changes in the GR and associated proteins (Pariante and Lightman, 2008; Miller and Raison, 2016). These modifications lead to a desensitization to cortisol (Herkenham and Kigar, 2017). Yet, one of the functions of glucocorticoids is to inhibit the peripheral immune response by inducing amongst other actions, immune cell apoptosis and suppression of NF-κB signaling, which is the main pathway responsible for the production of cytokines (Slavich and Irwin, 2014). These findings produce a seemingly paradoxical situation in which depression is characterized by high levels of both cortisol and proinflammatory factors. In animal models of glucocorticoid insensitivity, immune cells become desensitized to the immunosuppressing effects of glucocorticoids (Silverman and Sternberg, 2012), leading to higher levels of proinflammatory cytokines which further inhibit GR function (Slavich and Irwin, 2014; Miller and Raison, 2016). In addition, in rodents, GSK3, which is activated during depressive-like behaviors and which promotes proinflammatory cytokine production, also phosphorylates the GR, inhibiting its transcriptional activity (Rogatsky et al., 1998). Furthermore, GSK3 knockin mice, which have constitutively active GSK3, also exhibit increased levels of glucocorticoids after exposure to stress (Polter et al., 2010). GSK3 is degraded upon glucocorticoid signaling activation, leading to the formation of adherens junctions and increased tight junction strength (Failor et al., 2007), which might favor the immune suppressive actions of the glucocorticoids. Altogether, glucocorticoids are important immunoregulators.

#### Vagus Nerve

The vagus nerve is the tenth cranial nerve and is a complex nerve that innervates multiple organs through afferent and efferent fibers throughout the body (Carreno and Frazer, 2017). One major hub of vagal afferent termination is the nucleus tractus solitarius of the medulla which directly and indirectly innervates several regions of the brain associated with depression (e.g., limbic forebrain) and inhibits the HPA axis, reducing cortisol secretion (Ondicova et al., 2010). The vagus nerve functions as a brake slowing the heart, inhibiting the sympathetic and adrenal activity and ultimately reducing stress reactivity. The vagus nerve has been implicated in immune function. Thus, subdiaphragmatic vagal afferents provide information to the brain regarding visceral state. During inflammation with elevation of proinflammatry cytokines (IL-6, TNFα, IL-1β), the vagus nerve mediates in part the induction of sickness behavior (Dantzer et al., 2008), through immune cells (e.g., macrophages and dendritic cells) present in the perineural sheath of the vagus nerve (Dantzer, 2009) that relay the inflammatory signal to the brain. Consistent with this, cytokines elevated by LPS administration activate brain regions downstream of vagal afferent input (e.g., brainstem, hypothalamus, and limbic structures) and sectioning of the vagus nerve abolishes LPS-induced sickness behavior despite an elevation of cytokines (Pavlov and Tracey, 2012). This suggests that afferent terminals of the vagus nerve sense peripheral inflammation and affects depression-associated brain regions that may contribute to behavioral and neuroendocrine changes observed in MDD patients. Reciprocally, stimulation of the vagus nerve is associated with reduced production of proinflammatory cytokines (IL-6, TNFα, and others) (Bernik et al., 2002), which has been named the “inflammatory reflex.” The latter is mediated by cholinergic signaling and regulated by the afferent fibers of the vagus nerve (Pavlov and Tracey, 2012). Cholinergic signaling through α7-nicotinic acetylcholine receptors expressed on immune cells inhibits NF-κB-dependent proinflammatory cytokine (e.g., IL-1β and TNFα) secretion by macrophages, T cells, and dendritic cells (Pavlov and Tracey, 2012). These effects may contribute to the efficacy of chronic vagal nerve stimulation as a treatment in patients with treatment-resistant depression (Carreno and Frazer, 2017). Overall, the vagus nerve serves the brain-immune axis as a centrally controlled inflammation rheostat. GSK3 has been shown to mediate parasympathetic dysfunction in a diabetic mice model (Zhang et al., 2014), and to promote neuropathic pain, whereas GSK3 inhibition ameliorates neuropathic pain by reducing proinflammatory cytokines, and increasing serotonergic and catecholaminergic pathways (Mazzardo-Martins et al., 2012).

### Effects of Inflammation on Brain Functions

Neuroinflammation is a condition of elevated levels of pro-inflammatory cytokines in the CNS, and is induced in response to psychosocial stress as well as infection or injury (Rivest, 2009; Shatz, 2009; Benarroch, 2013; Le Thuc et al., 2015; Ransohoff et al., 2015). Acute neuroinflammation is often beneficial and neuroprotective following infection or injury of the CNS (Bitzer-Quintero and Gonzalez-Burgos, 2012; Le Thuc et al., 2015). For example, following injury peripheral immune cells, such as monocytes and lymphocytes (Stoll et al., 2002; Whitney et al., 2009), and microglia (Bitzer-Quintero and Gonzalez-Burgos, 2012), are recruited to the site of insult by chemokines (Ransohoff and Engelhardt, 2012). Once recruited, the cells act to resolve the insult in part by phagocytosing cellular debris caused by damage, and releasing anti-inflammatory cytokines and neurotrophic factors, promoting neurogenesis and synaptogenesis, overall fostering recovery after neuronal damage (Mathieu et al., 2010; Bitzer-Quintero and Gonzalez-Burgos, 2012; Le Thuc et al., 2015). However, excessive or prolonged neuroinflammation is detrimental to CNS functions, impairing learning and memory, inhibiting long-term potentiation, decreasing neurogenesis, and altering dendritic spine density (Bilbo and Schwarz, 2009), synaptic plasticity, and synaptic scaling (Jakubs et al., 2008; McAfoose and Baune, 2009; Wood et al., 2011; Chugh et al., 2013; Kohman and Rhodes, 2013; Green and Nolan, 2014; Vezzani and Viviani, 2015). Additionally, chronic neuroinflammation leads to decreased production of brain-derived neurotrophic factor (BDNF) by neurons and astrocytes which may impair neurogenesis in the dentate gyrus of the hippocampus (Sapolsky, 2001; Jo et al., 2015) and alter neuronal functional integration (Jakubs et al., 2008; Wood et al., 2011). Neuroinflammation increases the production of neurotoxic factors such as reactive oxygen species (Whitney et al., 2009) and glutamate leading to excitotoxicity, increases BBB permeability (Pan et al., 2011) allowing inappropriate peripheral immune cell infiltration into the CNS (Lossinsky and Shivers, 2004), and perpetuates further production of pro-inflammatory cytokines (Le Thuc et al., 2015). Altogether, these processes cause damage to neurons, impairing neurogenesis and healthy synaptic connectivity in both the adult (Das and Basu, 2008) and developing brain (Harry and Kraft, 2012). Overall, excessive or prolonged neuronflammation is associated with pathogenicity in multiple neurological diseases, including mood disorders (Borsini et al., 2015).

## Interactions Between Antidepressant Actions and the Immune Response Of Depression

### Effects of Antidepressants on Cytokine Levels in Depressed Patients and in Mouse Models of Depression

Major depressive disorder is associated with inflammation, and antidepressant treatments are generally thought to shift the balance toward anti-inflammatory responses (Sluzewska et al., 1997; Maes, 1999; Lanquillon et al., 2000; Kubera et al., 2001a) leading to an overall normalization of cytokine levels to healthy control levels (Maes et al., 1997; Tuglu et al., 2003; O’Brien et al., 2004; Howren et al., 2009; Hannestad et al., 2011). However, the role of antidepressant medications in regulating cytokine production differs according to the class of antidepressant used. Thus, selective serotonin reuptake inhibitors (SSRIs) seem to reduce IL-1β, IL-6, and TNFα, whereas serotonin and norepinephrine reuptake inhibitors (SNRIs) can have proinflammatory effects, increasing in particular TNFα and IL-6 levels (Piletz et al., 2009) consistent with the proinflammatory effects of norepinephrine on innate immune cells (Thayer and Sternberg, 2010). Moreover, sertraline (SSRI) therapy decreases IL-12 levels but increases IL-4 and TGFβ levels in responders (Sutcigil et al., 2008). Amitriptyline [tricyclic antidepressant (TCA)] therapy in MDD patients is associated with decreased TNFα (Hinze-Selch et al., 2000), whereas desipramine, increases IL-10 in MDD patients but does not affect pro-inflammatory cytokines (Roque et al., 2009). In addition to decrease proinflammatory cytokine levels, acute antidepressant treatment can increase anti-inflammatory cytokine levels (e.g., IL-1ra, IL-10, TGFβ) (Seidel et al., 1995, 1996a; Maes et al., 1999; Roumestan et al., 2007). In mice, antidepressants also inhibit LPS- (Ohgi et al., 2013) or cytokine-induced depressive-like behaviors (Yirmiya et al., 2001), whereas antidepressant medications have also been shown to increase proinflammatory cytokines in mice (Warner-Schmidt et al., 2011).

Conversely, blocking proinflammatory cytokines provides antidepressant effects. Thus, FDA-approved monoclonal antibodies and other cytokine inhibitors, approved for other inflammatory conditions, to block individual cytokines in MDD patients, provide significant antidepressant effects in some cohorts of MDD patients (Kappelmann et al., 2016). Thus, TNFα inhibitors such as adalimumab (Loftus et al., 2008; Menter et al., 2010), etanercept (Tyring et al., 2006, 2013), or infliximab (Raison et al., 2013); IL-12/IL-23 antagonists (Langley et al., 2010); IL-6 antagonists (Kappelmann et al., 2016); or IL-4Ra antagonists (Simpson et al., 2015) are more efficacious than placebo in the treatment of MDD. Taken together, modulation of pro-inflammatory and/or anti-inflammatory cytokines might provide antidepressant actions and antidepressant medications might shift the balance toward anti-inflammatory responses.

### Modulatory Effects of Cytokines on Therapeutic Responses to Antidepressants

Antidepressant resistance has often been associated with an activation of the inflammatory system. Thus, MDD patients displaying inflammation prior to treatment are less responsive to antidepressants (Mikova et al., 2001), or lithium (Sluzewska et al., 1997). Furthermore, IL-6 levels, but not TNFα, prior to treatment with amitriptyline predict the patient response (Lanquillon et al., 2000; Kubera et al., 2001a; Benedetti et al., 2002). Altogether, response to antidepressant treatments is impaired by certain proinflammatory cytokines, and can be overcome by co-administering anti-inflammatory drugs (Köhler et al., 2014). The results of a meta-analysis supported the notion that adjuvant anti-inflammatory therapy in addition to antidepressant treatment of depression can provide significant clinical benefit (Köhler et al., 2014). However, non-steroidal anti-inflammatory drugs (NSAIDs) might not be the optimal anti-inflammatory approach to use, as in the SADHART study, all patients receiving NSAIDs continued to exhibit cytokine elevations (Glassman et al., 2002). Recent findings point toward potential cytokine biomarker for antidepressant response. Thus, higher level of IL-17A predicts better outcomes in the response to bupropion and SSRI combination therapy (Jha et al., 2017). Furthermore, levels of CRP predict treatment response differentially according to the antidepressant. Thus, low level of CRP predicts better response to escitalopram, whereas high level CRP is associated with better outcome with nortriptyline (Uher et al., 2014). Overall these findings suggest that cytokine levels may influence a patient’s response to antidepressant treatment.

## Conclusion

Inflammation, mainly cytokine production, has become an accepted part of the etiology of MDD, however, the role of the cytokines in promoting depression remains unclear. Targeting cytokines seems to provide some clinical benefits, but unless the immune response is better characterized in MDD patients, attenuating cytokine production in MDD patients might remain challenging.

## Author Contributions

EM-R, JL, RW, SS, and EB wrote the review.

## Conflict of Interest Statement

The authors declare that the research was conducted in the absence of any commercial or financial relationships that could be construed as a potential conflict of interest.
